# Optical gain in 1.3-μm electrically driven dilute nitride VCSOAs

**DOI:** 10.1186/1556-276X-9-22

**Published:** 2014-01-13

**Authors:** Sefer Bora Lisesivdin, Nadir Ali Khan, Simone Mazzucato, Naci Balkan, Michael John Adams, Ville-Markus Korpijärvi, Mircea Guina, Gabor Mezosi, Marc Sorel

**Affiliations:** 1 School of Computer Science and Electronic Engineering, University of Essex, Wivenhoe Park CO4 3SQ, UK; 2 Department of Physics, Faculty of Science and Arts, Gazi University, Teknikokullar, Ankara 06500, Turkey; 3 Optoelectronics Research Centre (ORC), Tampere University of Technology, P.O. Box 692, Tampere 33101, Finland; 4 School of Engineering, University of Glasgow, Glasgow GG12 8QQ, UK; 5 Kohat University of Science and Technology (KUST), Kohat, Khyber Pakhtoonkhwa, Pakistan

**Keywords:** Dilute nitride, Optical injection, Gain, Vertical cavity semiconductor optical amplifiers

## Abstract

We report the observation of room-temperature optical gain at 1.3 μm in electrically driven dilute nitride vertical cavity semiconductor optical amplifiers. The gain is calculated with respect to injected power for samples with and without a confinement aperture. At lower injected powers, a gain of almost 10 dB is observed in both samples. At injection powers over 5 nW, the gain is observed to decrease. For nearly all investigated power levels, the sample with confinement aperture gives slightly higher gain.

## Background

In_*x*_Ga_1-*x*_As_1-*y*_N_*y*_ semiconductor alloy was first proposed by Kondow et al. in 1996 [1], and considerable research attention has been devoted to this alloy system due to its possible optoelectronic applications at an operating wavelength of 1.3 μm. With the addition of small amounts of nitrogen into the (In)GaAs lattice, a strong electron confinement and bandgap reduction are obtained. Furthermore, addition of N allows band engineering, allowing the device operating wavelength range to extend up to 1.6 μm [2]. An extensive set of different devices based on this alloy has been fabricated and demonstrated [3]. Examples of these devices are vertical cavity surface-emitting lasers (VCSELs) [4-6], vertical external cavity surface-emitting lasers [7,8], solar cells [8,9], edge-emitting lasers [10], photodetectors [11], semiconductor optical amplifiers (SOAs) [12], and vertical cavity semiconductor optical amplifiers (VCSOAs) [13,14].

VCSOAs can be seen as the natural evolution of SOAs, which, owing to their fast response, reduced size, and low-threshold nonlinear behavior, are popular in applications such as optical routing, signal regeneration, and wavelength shifting. Within these fields, VCSOAs have been used as optical preamplifiers, switches, and interconnects [15-17]. Their geometry provides numerous advantages over the edge-emitting counterpart SOAs, including low noise figure, circular emission, polarization insensitivity, possibility to build high-density two-dimensional arrays of devices that are easy to test on wafer, and low-power consumption that is instrumental for high-density photonic integrated circuits. Generally speaking, a VCSOA is a modified version of a VCSEL that is driven below lasing threshold. The first experimental study of an In_*x*_Ga_1-*x*_As_1-*y*_N_*y*_/GaAs-based VCSOA was reported in 2002 [18], with a theoretical analysis published in 2004 [19]. Several studies on optically pumped In_*x*_Ga_1-*x*_As_1-*y*_N_*y*_ VCSOAs have been published [14,20-23], while electrically driven VCSOAs have been demonstrated only in ‘Hellish’ configuration [24]. The present contribution builds on these technological developments to focus on an electrically driven multifunction standard VCSOA device operating in the 1.3-μm wavelength window.

## Methods

**Figure 1:**
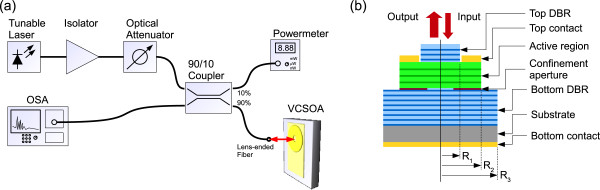
Experimental setup (a) and the layer structure of the investigated samples (b).

The amplification properties of In_*x*_Ga_1-*x*_As_1-*y*_N_*y*_ VCSOAs were studied using a 1,265- to 1,345-nm tunable laser (TL; TLM-8700-H-O, Newport Corporation, Irvine, CA, USA), whose output was sent to the sample using the setup shown in Figure 1a. The TL signal was split via a 10/90 coupler to a power meter and to the sample, respectively. Back reflections were avoided using an optical isolator while the TL power was changed from 0 to 7 mW using an optical attenuator. A lens-ended fiber (SMF-28 fiber, conical lens with cone angle of 80° to 90° and radius of 6.0 ±1.0 μm) was used to focus the TL light to the sample surface as well as to collect its reflected/emitted/amplified light, which was then directed to an optical spectrum analyzer (OSA). The VCSOA was electrically DC biased up to 10 mA and stabilized in temperature at 20°C via a Peltier cooler.

The investigated VCSOA structure with a 3.5λ cavity is shown in Figure 1b. The structures were grown by a solid source molecular beam epitaxy reactor with a radio frequency plasma source for incorporating nitrogen. The growth was carried on an n-type GaAs(100) substrate, and the bottom and top distributed Bragg reflectors (DBRs) were doped with silicon (n-type) and beryllium (p-type), respectively. The two DBRs comprised 21 and 24 pairs of Al_*x*_Ga_1-*x*_As/GaAs layers for the top and bottom DBR, respectively. The Al concentrations were *x* = 0.8 and 0.98 in the top and bottom DBRs, respectively. The confinement aperture, which is required for better carrier and light confinement, was defined in the uppermost layer of the bottom DBR. The active region contains three stacks of three 7-nm-thick In_0.35_Ga_0.65_As_0.975_ N_0.025_ quantum wells separated by 20-nm thick GaAs spacers. A set of several VCSOA samples was fabricated, having different dimensions of the top DBR mirror radius (*R*_1_), confinement aperture radius (*R*_2_), and bottom DBR radius (*R*_3_) for cases with and without the confinement aperture. In this paper, we compare the results obtained for two samples with and without confinement aperture, with *R*_1_ = 5 μm, *R*_2_ = 25 μm, and *R*_3_ = 50 μm.

## Results and discussion

**Figure 2:**
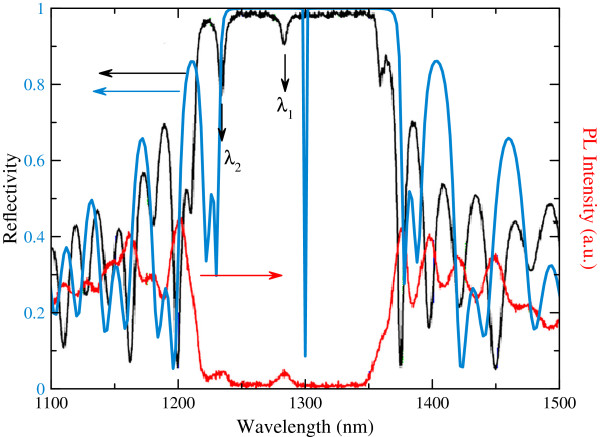
**Room temperature photoluminescence (red) and reflectance spectra of the studied structure.** Experimental and simulated reflectivity spectra of the studied VCSOA structure are shown in black and blue lines, respectively.

**Figure 3:**
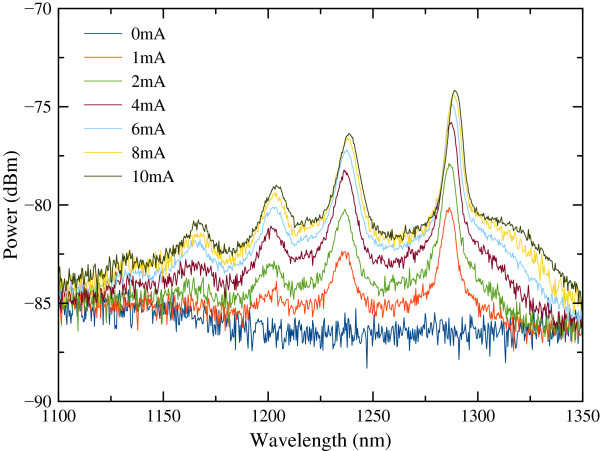
Power spectra of VCSOA without confinement aperture obtained for different bias currents.

Room-temperature reflectivity and photoluminescence (PL) measurements were performed on the as-grown sample, and the results are shown in Figure 2. Simulated reflection is also shown in the figure. Two resonances *λ*_R1_ and *λ*_R2_ are observed within the DBR stop band as a result of the relatively long cavity length [25]. The principle resonance, which is designed for 1.3-μm operation, is observed at *λ*_R1_ = 1,282 nm, while the other unwanted resonance at lower wavelength is observed at *λ*_R2_ = 1,235 nm. Figure 3 shows the VCSOA amplified spontaneous emission (ASE) spectra obtained with no optical injection at different applied bias currents of 0 to 10 mA for the sample without confinement aperture. The highest ASE power peak appears at 1,288 nm and is blue-shifted with respect to that of the lasing cavity mode wavelength [26,27]. The other modes are also consistent with the PL spectra. Figure 3 shows that with increasing the bias current, the amplitude of each mode increases and also slightly shifts towards higher wavelengths. This shift is associated with local temperature increase in the device. A similar result was observed in the VCSOA with the confinement aperture.

**Figure 4:**
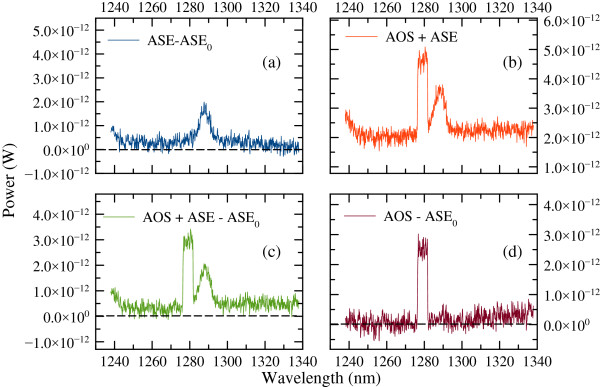
**Results of various power spectra for λ**_**inj **_**= 1,279 nm. (a)** ASE - ASE_0_level, **(b)** AOS + ASE, **(c)** AOS + ASE - ASE_0_, and **(d)** AOS - ASE_0_ power spectra.

Since no significant change in the spectrum amplitude above 7 mA was observed, we investigated the devices up to this current value. ASE power spectra taken at 7 and 0 mA (ASE_0_) are used to extract the spectrum ASE - ASE_0_, which is the spectrum where the background noise is minimized. To find the amplified optical signal (AOS), we injected light sweeping the TL wavelength (*λ*_inj_) from 1,266 to 1,310 nm with a 7-mA current bias. Figure 4 shows results for injection at *λ*_inj_ =1,279 nm only. We could not investigate the second resonance peak *λ*_R2_ because of the wavelength limit of the TL. In Figure 4a,b,c,d, the results for ASE - ASE_0_, AOS + ASE, AOS + ASE - ASE_0_, and finally AOS - ASE_0_spectra are shown, respectively.

**Figure 5:**
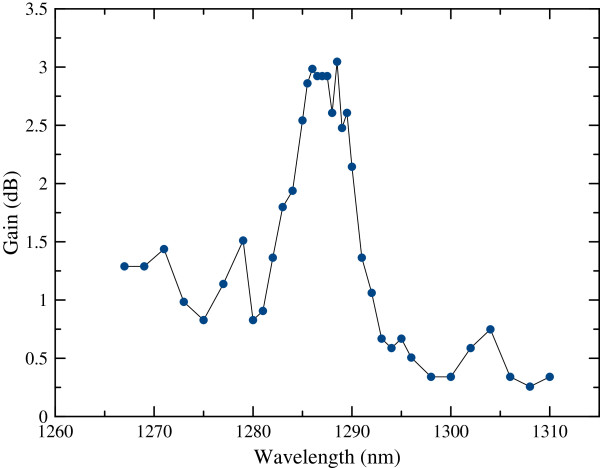
**Gain versus injected laser wavelength with *P***_**inj **_**= 2.25 nW.**

As the gain is small, the amplified signal cannot be easily discerned in Figure 4d. Hence, the gain was calculated using the simple relation

(1) $$\mathrm{Gain}=\frac{\mathrm{AOS}+\mathrm{ASE}‒\mathrm{AS}E_{0}}{\mathrm{AOS}‒\mathrm{AS}E_{0}}$$

for each wavelength after obtaining AOS and ASE data. Results are shown as a function of the injected wavelength in Figure 5 for a specific laser power (*P*_inj_) of 2.25 nW. A maximum gain of 3 dB with a very broad peak is observed at the maximum ASE wavelength of 1,288.5 nm. In the study, measured signal levels are very near to limits of the OSA; therefore, larger bandwidth wavelength values are used, which can be the reason of the broadness of the gain peak.

**Figure 6:**
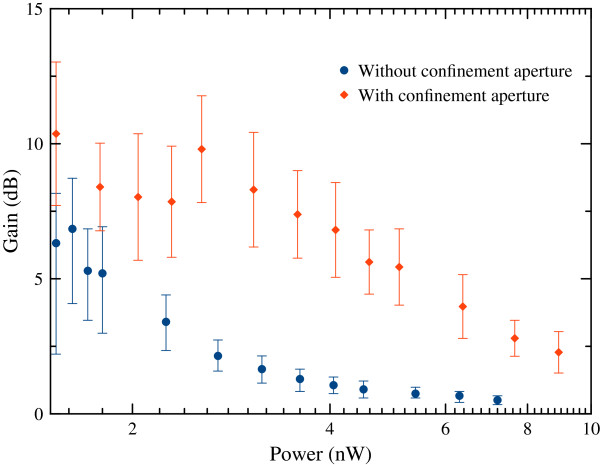
Power-dependent gain for the samples with and without confinement aperture.

Having verified that the gain peak corresponds to the ASE peak wavelength, we investigated the *P*_inj_ dependence by varying it from 1.5 nW to a few milliwatts for the single wavelength of 1,288.5 nm. Results are presented for both samples with and without confinement aperture in Figure 6 for power values below 10 nW. For injected laser powers over 5 nW, the gain falls rapidly. At the lowest injected power, the sample with confinement aperture exhibits 10 dB of gain, which is observed near the maximum ASE wavelength. For the investigated injected power range, the sample with the confinement aperture showed a higher gain because of the better carrier and light confinement in the VCSOA.

## Conclusions

In this paper, we report the observation of gain in an electrically driven dilute nitride VCSOA device operated at 1.3-μm in reflection mode. Two different types of samples with and without confinement aperture are investigated. The ASE power peak is found to be at 1,288.5 nm with additional modes, which are caused by the length of the cavity. Optical gain is found to occur at low optical injection values. Above 5 nW of optical injection, the gain is found to fall rapidly. The maximum observed optical gain is observed at 1,288.5 nm at room temperature. The maximum observed optical gain at 7-mA current at room temperature is around 10 and 6 dB for samples with and without confinement aperture, respectively. It is important to mention that despite the small gain, the device is very promising because it requires very small currents compared with in-plane SOAs.

## Competing interests

The authors declare that they have no competing interests.

## Authors’ contributions

SBL, NAK, and SM carried out the measurements and data analysis. VMK and MG performed the growth of structures. GM and MS carried the fabrication of devices. MJA performed the theoretical studies and analysis. NB is the project leader. SBL and NB wrote the paper. All authors read, corrected, and approved the final manuscript.
